# Supplementary figures and images for: RecombiCraft library construction: A novel method for DNA library cloning and expansion using non-enzymatic single-step DNA recombination and liquid culture
Source: PLoS One. 2024 Dec 2;19(12):e0312188. doi: 10.1371/journal.pone.0312188 (PMC11611171; doi:10.1371/journal.pone.0312188)

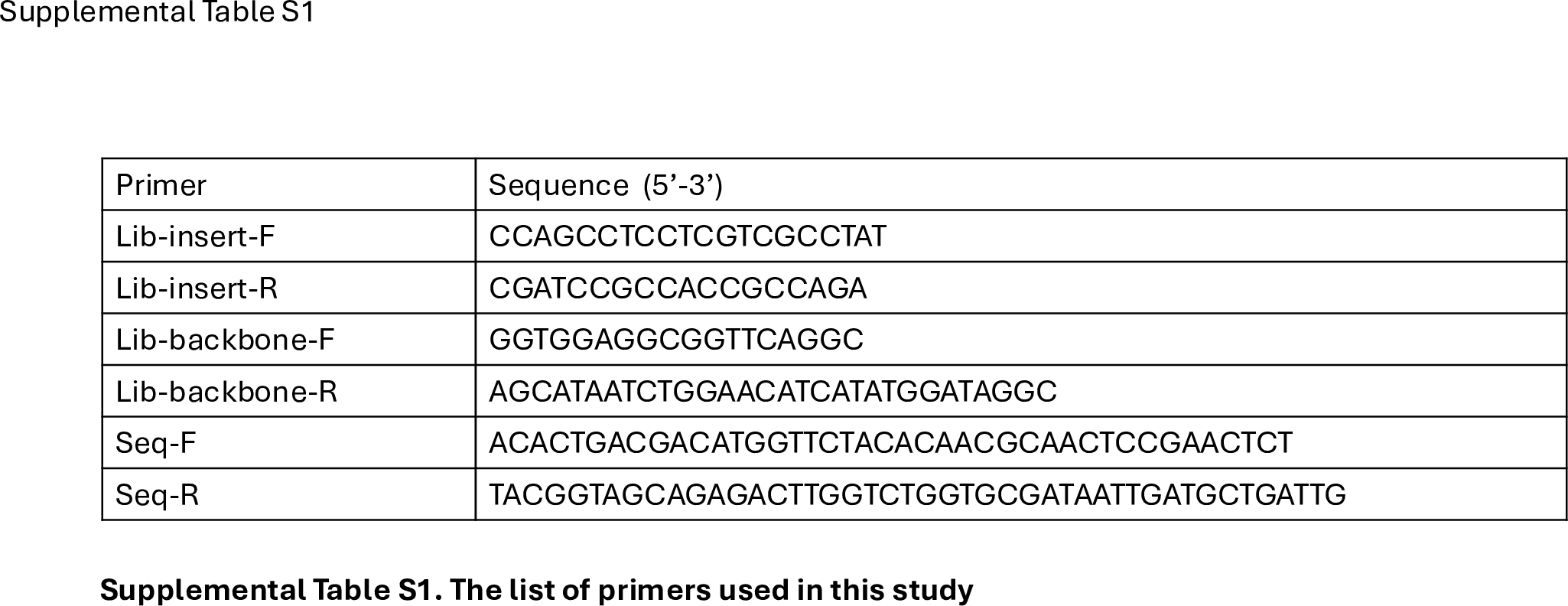

Supplement: S1 Table — (TIF) [file pone.0312188.s001.tif]

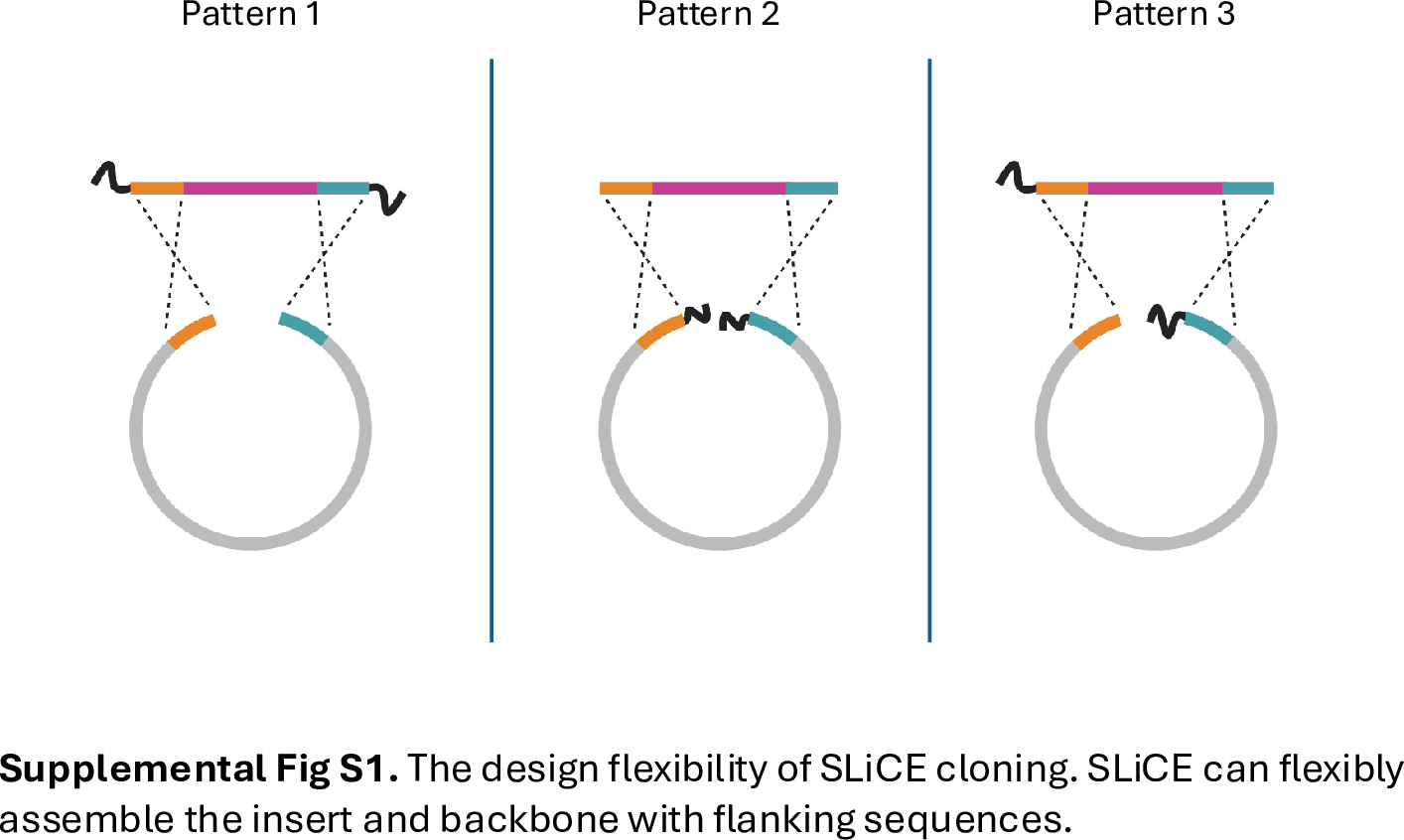

Supplement: S1 Fig — (TIF) [file pone.0312188.s002.tif]
