# Supplementary material for: RecombiCraft library construction: A novel method for DNA library cloning and expansion using non-enzymatic single-step DNA recombination and liquid culture
Source: PLoS One. 2024 Dec 2;19(12):e0312188. doi: 10.1371/journal.pone.0312188 (PMC11611171; doi:10.1371/journal.pone.0312188)
Supplement: S1 Raw images — (PDF) [file pone.0312188.s003.pdf]

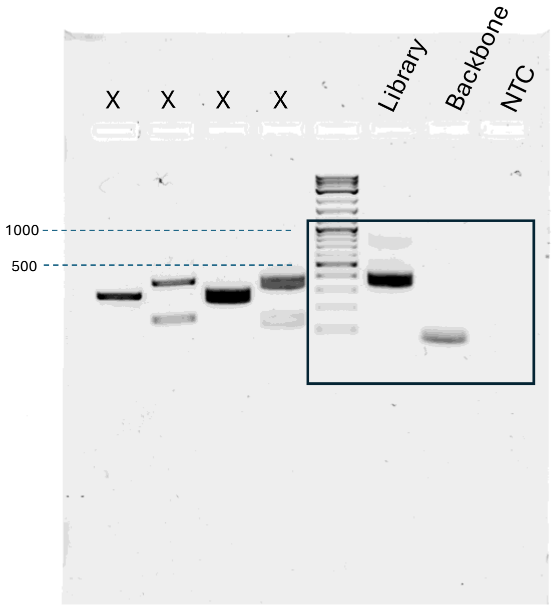

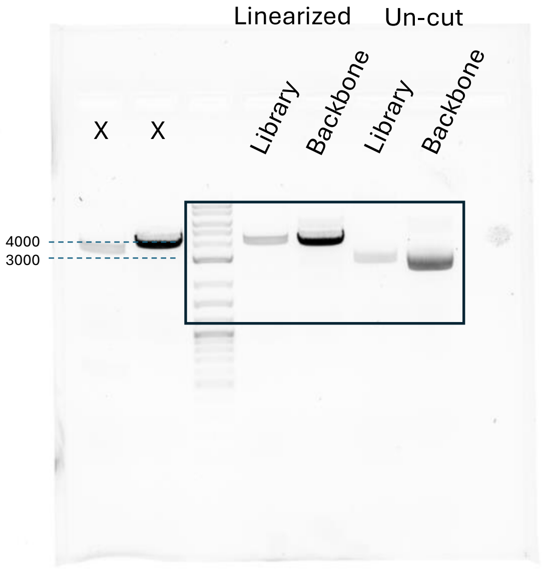

S1. Raw image of figure 3

The image was taken by ChemiDoc MP (BIO-RAD). The black square is used for the figure in the manuscript.
