# Supplementary material for: RecombiCraft library construction: A novel method for DNA library cloning and expansion using non-enzymatic single-step DNA recombination and liquid culture
Source: PLoS One. 2024 Dec 2;19(12):e0312188. doi: 10.1371/journal.pone.0312188 (PMC11611171; doi:10.1371/journal.pone.0312188)
Supplement: Attachment — Submitted filename: Responses.docx [file pone.0312188.s004.docx]

We appreciate the positive response from the editor/reviewer. The manuscript has been edited according to the suggestions. Point-by-point response is as follows:

Reviewer 1

Major concern
1. The author introduces RecombiCraft as an "innovative, rapid, and cost-efficient method" but doesn't provide enough specific details on what makes it unique compared to existing methods. Adding data or results could strengthen the claim.
>Thank you for your comment. We added the sentence to emphasize uniqueness in the abstract. Figure 4 also explains the difference between other traditional methods.

Query 2: The manuscript would be significantly strengthened if you will do comet assay to check the DNA integrity after exposure and recovery of crude oil.
>Thank you for your comment. This method proposes DNA library preparation, we think that figure 3 shows purity good enough for downstream applications.

Query 3: The quality of some figures is not adequate for publication.
>We have separately uploaded high-resolution images as figures.

Query 4: In discussion section, rephrased some sentences for better readability and coherence.
>We reassessed these sections and have modified the text to improve readability.

Query 5: Made the comparison between SLiCE cloning and traditional methods clearer and more concise.

>We have included additional text within the Discussion section to emphasize the superior aspects of this method.

Minor comments:
Line 27: The phrase "significantly shorter library generation period" is vague. Providing specific time comparisons with other methods would make this claim more impactful.

>We have now specified the minimum time to prepare DNA library in the Abstract.

Line 102: Library DNA" should either both be capitalized and not, depending on whether it's a proper noun. It might be better as "library DNA". Please check it throughout the manuscript.

> All spellings except the first word have been unified to "library."

Line 116-117: The sentence "To optimize library cloning, we created homologous sequences of varying lengths and compared their cloning efficiency." could be simplified or rephrased for clarity.

>This sentence has been rephrased: “We improved library cloning by creating different-length homologous sequences and comparing their cloning efficiency.“

Line 149-151: Please omit correct the sentence in following ways.

The monobody library was designed based on a hydrophilic variant of human fibronectin, which improves biodistribution properties, making it well-suited for downstream applications in molecular imaging and therapeutics days post fertilization as you mentioned it before.

> The statement has been corrected as indicated.

Line 152: The term "throughout" is omitted, which makes the sentence slightly redundant. Correct the sentence in following ways.

This hydrophilic monobody library included diverse amino acid sequences in three solvent-exposed binding loops, akin to antibody CDR-H1/2/3, while conserving amino acids in the framework positions.

> The statement has been corrected as indicated.

Line 159: The phrase "at least backbone" is confusing and seems incorrect.

>”at least” was removed to correct this sentence.

Line 164-165: he phrase "not only... but also" suggests two parallel ideas, but the way it's phrased here makes it slightly unclear. Correct the sentence in following ways.

This optimized construction method confirmed the absence of unwanted sequences and validated the structural integrity and diversity of the monobody library.

> The statement has been corrected as indicated.

Line 172: Used "host cells" instead of "suitable cells" for clarity and consistency.

> The statement has been corrected as indicated.

Line 192: Replaced "however" with a semicolon to improve readability and flow.

> The statement has been corrected as indicated.

Line 198: Used "may" instead of "might" for a more formal tone and added "generally" to clarify that the correlation is not absolute.

> The statement has been corrected as indicated.

Line 220-223: Changed "named RecombiCraft" to "which we have named RecombiCraft" for clarity. Changed "enables acquisition of sufficient DNA quantities" to "facilitates the acquisition of sufficient DNA quantities" for smoother readability. Changed "fine-tuning" to "optimizing" for a more formal tone and clearer meaning.

> The statement has been corrected as indicated.

Reviewer 2

Thank you very much for the positive comments.

We have uploaded a higher-resolution image tiff file.
